# Supplementary material for: Association of ankle-brachial index with cognitive decline in patients with lacunar infarction
Source: PLoS One. 2022 Feb 4;17(2):e0263525. doi: 10.1371/journal.pone.0263525 (PMC8815973; doi:10.1371/journal.pone.0263525)
Supplement: S1 Table — (DOCX) [file pone.0263525.s001.docx]

**S1 Table. SBP and DBP of the four limbs and bilateral ankle-brachial pressure index and brachial-ankle pulse wave velocity**

|  | n=176 |
| --- | --- |
| SBP (left upper limb), mmHg | 150.2±21.0 |
| DBP (left upper limb), mmHg | 85.8±12.7 |
| SBP (right upper limb), mmHg | 151.2±19.7 |
| DBP (right upper limb), mmHg | 86.0±11.7 |
| SBP (left lower limb), mmHg | 172.9±25.2 |
| DBP (left lower limb), mmHg | 85.1±14.0 |
| SBP (right lower limb), mmHg | 172.9±27.1 |
| DBP (right lower limb), mmHg | 85.9±15.0 |
| Ankle brachial pressure index (left) | 1.13±0.10 |
| Ankle brachial pressure index (right) | 1.13±0.11 |
| Brachial-ankle pulse wave velocity (left) | 2083.2±550.1 |
| Brachial-ankle pulse wave velocity (right) | 2089.2±563.1 |

SBP, systolic blood pressure; DBP, diastolic blood pressure

Data are presented as mean±standard deviation
