# Supplementary material for: Association of ankle-brachial index with cognitive decline in patients with lacunar infarction
Source: PLoS One. 2022 Feb 4;17(2):e0263525. doi: 10.1371/journal.pone.0263525 (PMC8815973; doi:10.1371/journal.pone.0263525)
Supplement: S2 Table — (DOCX) [file pone.0263525.s002.docx]

**S2 Table.** **Characteristics of each group**

|  | Group 1 ABI≥1.0 baPWV≤2019 cm/s n=78 | Group 2 ABI≥1.0 baPWV>2019 cm/s n=79 | Group 3 ABI<1.0 baPWV≤2019 cm/s n=10 | Group 4 ABI<1.0 baPWV>2019 cm/s n=9 | p value |
| --- | --- | --- | --- | --- | --- |
| Age, year | 67.7±10.8 | 75.9±8.9 | 73.8±17.2 | 83.3±11.3 | <0.001 |
| Sex (female), n (%) | 25 (32.1) | 31 (39.2) | 5 (50.0) | 6 (66.7) | <0.001 |
| Body mass index, kg/m^2^ | 24.1±3.2 | 23.7±3.9 | 24.4±6.3 | 22.4±2.5 | 0.521 |
| Education, year | 13.4±2.0 | 12.1±2.4 | 10.5±2.1 | 9.3±1.8 | <0.001 |
| MMSE score, median (IQR) | 28 (26-29) | 27 (26-29) | 26 (24.5-29) | 25 (22.5-27.5) | 0.021 |
| Hypertension, n (%) | 50 (64.1) | 59 (74.7) | 6 (60.0) | 9 (100.0) | 0.090 |
| Diabetes mellitus, n (%) | 18 (23.1) | 23 (29.1) | 1 (10.0) | 2 (22.2) | 0.551 |
| Dyslipidemia, n (%) | 44 (56.4) | 46 (58.2) | 5 (50.0) | 6 (66.7) | 0.898 |
| Chronic kidney disease, n (%) | 17 (21.8) | 22 (27.9) | 1 (10.0) | 5 (55.6) | 0.097 |
| Current smoker, n (%) | 31 (39.7) | 27 (34.2) | 5 (50.0) | 1 (11.1) | 0.280 |
| Habitual drinker, n (%) | 35 (44.9) | 34 (43.0) | 1 (10.0) | 2 (22.2) | 0.116 |
| Antihypertensive drug, n (%) | 42 (53.9) | 58 (73.4) | 4 (40.0) | 7 (77.8) | 0.022 |
| Antidiabetic drug, n (%) | 14 (18.0) | 17 (21.5) | 0 (0) | 0 (0) | 0.179 |
| NIHSS score, median (IQR) | 2 (1, 3) | 1 (1, 3) | 1.5 (0, 3.25) | 2 (0.5, 3) | 0.950 |
| Location of infarction |  |  |  |  |  |
| Corona radiata, n(%) | 16 (20.5) | 28 (35.4) | 5 (50.0) | 3 (33.3) | 0.092 |
| Basal ganglia, n(%) | 4 (5.1) | 5 (6.3) | 0 (0) | 1 (11.1) | 0.752 |
| Capsulae internae, n(%) | 17 (21.8) | 18 (22.8) | 1 (10.0) | 2 (22.2) | 0.834 |
| Thalamus, n(%) | 26 (33.3) | 21 (26.6) | 1 (10.0) | 2 (22.2) | 0.408 |
| Brain stem, n(%) | 15 (19.2) | 7 (8.9) | 3 (30.0) | 1 (11.1) | 0.147 |
| MRI findings |  |  |  |  |  |
| DSWMH, median (IQR) | 1 (1, 2) | 2 (1, 2) | 1 (1, 1.25) | 1 (1, 2) | 0.035 |
| PVH, median (IQR) | 1 (1, 2) | 2 (1, 2) | 1.5 (1, 2) | 2 (1.5, 2) | 0.086 |

ABI, ankle-brachial pressure index; baPWV, brachial-ankle pulse wave velocity; MMSE, Mini-Mental State Examination; IQR, interquartile range; NIHSS, National Institutes of Health Stroke Scale; MRI, magnetic resonance imaging; DSWMH, deep and subcortical white matter hyperintensity; PVH, periventricular hyperintensity

Data are presented as mean±standard deviation, median (25% IQR to 75% IQR), or the number of patients (%).
