# Supplementary material for: Association of ankle-brachial index with cognitive decline in patients with lacunar infarction
Source: PLoS One. 2022 Feb 4;17(2):e0263525. doi: 10.1371/journal.pone.0263525 (PMC8815973; doi:10.1371/journal.pone.0263525)
Supplement: S3 Table — (DOCX) [file pone.0263525.s003.docx]

**S3 Table.** **Background characteristics of the patients, including the patients with cerebral microbleeds and severe white matter lesions**

|  | n=268 |
| --- | --- |
| Age, year | 72.0±11.2 |
| Sex (female), n (%) | 93 (34.7) |
| Body mass index, kg/m^2^ | 23.8±3.6 |
| Education, year | 12.3±2.4 |
| MMSE score, median (IQR) | 27 (25-29) |
| Hypertension, n (%) | 203 (75.7) |
| Diabetes mellitus, n (%) | 67 (25.0) |
| Dyslipidemia, n (%) | 149 (55.6) |
| Chronic kidney disease, n (%) | 80 (29.9) |
| Current smoker, n (%) | 102 (38.1) |
| Habitual drinker, n (%) | 109 (40.7) |
| Antihypertensive drug, n (%) | 185 (69.0) |
| Antidiabetic drug, n (%) | 52 (19.4) |
| NIHSS score, median (IQR) | 2 (1, 3) |
| Location of infarction |  |
| Side of the lesion (left), n (%) | 140 (52.2) |
| Corona radiata, n(%) | 84 (31.3) |
| Basal ganglia, n(%) | 19 (7.1) |
| Capsulae internae, n(%) | 55 (20.5) |
| Thalamus, n(%) | 65 (24.3) |
| Brain stem, n(%) | 45 (16.8) |
| MRI findings |  |
| Cerebral microbleeds, n(%) | 71 (26.5) |
| DSWMH, median (IQR) | 2 (1, 2) |
| PVH, median (IQR) | 2 (1, 2) |
| Ankle brachial pressure index | 1.09±0.11 |
| Ankle brachial pressure index <1.0, n (%) | 37 (13.8) |
| Brachial-ankle pulse wave velocity, cm/s | 2181.3±573.5 |

MMSE, Mini-Mental Scale Examination; IQR, interquartile range; NIHSS, National Institutes of Health Stroke Scale; MRI, magnetic resonance imaging; DSWMH, deep and subcortical white matter hyperintensity; PVH, periventricular hyperintensity
