# Supplementary material for: Association of ankle-brachial index with cognitive decline in patients with lacunar infarction
Source: PLoS One. 2022 Feb 4;17(2):e0263525. doi: 10.1371/journal.pone.0263525 (PMC8815973; doi:10.1371/journal.pone.0263525)
Supplement: S4 Table — (DOCX) [file pone.0263525.s004.docx]

**S4 Table.** **Associations between multiple factors and decrease in MMSE scores in all patients, including the patients with cerebral microbleeds and severe white matter lesions (n=268)**

|  | Univariate analysis | | Multivariate analysis | |
| --- | --- | --- | --- | --- |
|  | Predictive value | p value | Predictive value | p value |
| Age | -0.054 | <0.001 | -0.024 | 0.145 |
| Sex (female) | -0.145 | 0.400 |  |  |
| Body mass index | 0.161 | <0.001 | 0.118 | 0.009* |
| Education | 0.209 | 0.002 | 0.022 | 0.766 |
| Hypertension | -0.145 | 0.448 |  |  |
| Diabetes mellitus | -0.224 | 0.237 |  |  |
| Dyslipidemia | 0.270 | 0.102 |  |  |
| Chronic kidney disease | -0.562 | 0.002 | -0.273 | 0.120 |
| Current smoker | -0.138 | 0.414 |  |  |
| Habitual drinker | -0.234 | 0.161 |  |  |
| Antihypertensive drug | -0.006 | 0.972 |  |  |
| Antidiabetic drug | -0.189 | 0.362 |  |  |
| NIHSS score | -0.111 | 0.290 |  |  |
| Location of infarction |  |  |  |  |
| Side of the lesion (left) | -0.085 | 0.606 |  |  |
| Corona radiata | 0.101 | 0.570 |  |  |
| Basal ganglia | -0.230 | 0.471 |  |  |
| Capsulae internae | 0.033 | 0.873 |  |  |
| Thalamus | -0.196 | 0.306 |  |  |
| Brain stem | 0.173 | 0.430 |  |  |
| MRI findings |  |  |  |  |
| Cerebral microbleeds | -0.566 | 0.002 | -0.357 | 0.060 |
| DSWMH, median | -0.310 | 0.137 |  |  |
| PVH, median | -0.842 | <0.001 | -0.381 | 0.085 |
| Ankle brachial pressure index <1.0 | -0.766 | 0.001 | -0.542 | 0.019* |
| Brachial-ankle pulse wave velocity >2086.5 cm/s | -0.261 | 0.111 |  |  |

MMSE, Mini-Mental Scale Examination; NIHSS, National Institutes of Health Stroke Scale; MRI, magnetic resonance imaging; DSWMH, deep and subcortical white matter hyperintensity; PVH, periventricular hyperintensity. * indicates <0.05
