# Supplementary material for: Association of ankle-brachial index with cognitive decline in patients with lacunar infarction
Source: PLoS One. 2022 Feb 4;17(2):e0263525. doi: 10.1371/journal.pone.0263525 (PMC8815973; doi:10.1371/journal.pone.0263525)
Supplement: Attachment — Submitted filename: response_to_reviewrs_R2_submission.docx [file pone.0263525.s007.docx]

Thank you for reviewing our manuscript. We appreciate your comments and suggestions. We have revised the manuscript in accordance with your comments. Our point-by-point responses to the comments are presented below.

**Response to the reviewers**

**Reviewer #1:** This manuscript explores the relationship between ABI and baPWV and cognition, as assessed by MMSE, in patients suffering from lacunar stroke. Patients with significant microbleeds and SVD were excluded from the analysis. Data show that ABI, but not baPWV is associated with lower overall cognition, orientation and immediate recall.
This is an interesting and well-written study investigating these relationships in a unique population. My main concern is that the numerous exclusion criteria resulted in a more limited sample size, especially for the low ABI groups, which were of most interest in this study. Major and minor concerns about this study are detailed below:

**Response:** We appreciate your insightful comments and advice. To address the main concern raised by you, we reanalyzed after adding the patients who were excluded based on MRI findings. We have responded to each of your comments below and denoted the corresponding revisions in the manuscript in red color.

**Comment 1:** The authors used numerous exclusion criteria to define their group, resulting in a limited sample of low ABI patients. This is unfortunate as their main results are in this group, but could also be driven by a number of other variable of interest which differs in this group. The authors should therefore better justify their choice of population, since this population is also not representative of the general population (since they all have a stroke).

**Response 1:** We appreciate your insightful comment. We tried to include patients with lacunar infarction and exclude patients with other etiologies. In fact, some cerebral microbleeds are associated with amyloid pathology, and some white matter lesions are associated with neurodegenerative diseases. However, cerebral microbleeds and white matter lesions are associated with small vessel diseases such as lacunar infarction. I also understand the critical point that you mentioned. To justify patient selection and perform comprehensive analyses, we added and analyzed the patients with CMBs and severe white matter lesions (who were excluded based on MRI findings) as well. The analyses revealed that low ABI was independently associated with the MMSE score. We have revised the manuscript and added the new results in the Supplemental Tables.

Lines 243-251

Finally, we added the patients with CMBs and severe white matter lesions (who were excluded based on MRI findings) and reanalyzed again (n=268). S3 shows the background characteristics of these patients. The median baPWV was 2086.5 cm/s and we divided the patients based on the median baPWV. We investigated the association of the MMSE score with the factors listed in S3. Univariate analysis revealed that the MMSE score was associated with age, BMI, education, chronic kidney disease, cerebral microbleeds, PVH, and ABI < 1.0 (p < 0.10), but not with baPWV > 2086.5 cm/s. Multivariate analysis revealed an independent association of BMI (p = 0.009) and ABI < 1.0 (p = 0.019) with the MMSE score (S4).

**Comment 2:** Field strength/MRI model was not taken into account in the analyses as a covariate of no interest. This should be done, especially if the distribution of patients scanned on each model is not equal amongst the different groups.

**Response 2:** Owing to the appointments in our institution, we used 1.5T MRI from July 1, 2011, and 3.0T MRI from April 2015 to December 31, 2018. This was incidental, not intentional. The imaging protocols were the same for the two groups. We investigated the differences in background characteristics between the two groups and found no significant differences. We have added the following text:

Lines 101-103

The imaging protocols were the same for the two MRI groups. We investigated the differences in background characteristics between the two groups and found no significant differences.

**Comment 3:** Was multiple comparison correction used for the analyses presented? Several comparisons are made and should therefore be corrected for.

**Response 3:** For multiple comparisons, we performed Bonferroni correction and Tukey’s honestly significant difference (HSD) test. We have revised the manuscript (Methods and Results section).

Lines 146-148

For multiple comparisons, the data were analyzed using one-way analysis of variance (ANOVA), and Bonferroni correction and Tukey’s honestly significant difference (HSD) test were performed.

Lines 201-203

Bonferroni correction and the HSD test revealed that the MMSE scores for the group with ABI < 1.0 and baPWV > 2019 cm/s were significantly lower than those for the group with ABI ≥ 1.0 and baPWV ≤ 2019 cm/s.

**Comment 4:** Since other aspects of health could explain some of the results shown for the four sub-groups, the demographic table should be expanded to include characteristics of the overall sample as shown currently, as well as for the four subgroups, rather than including this information in the supplemental materials.

**Response 4:** We have added the clinical information in Table 1.

**Comment 5:** Was Fazekas score, or better, WMH volume used in any of the analyses. Since WMH is likely to be related to cognition, it would be pertinent to use this in analyses as it could independently explain some of the variance in MMSE score.

**Response 5:** We added the data pertaining to CMBs and the Fazakas score and analyzed. We have revised the manuscript and tables accordingly.

**Comment 6:** The sample size in two of the groups is very small, potentially biasing some of the results. This should be acknowledged in the discussion.

**Response 6:** Thank you for this pointing this out. Per your suggestion, we have mentioned it as a limitation in the Discussion.

Lines 308-311

This study has several limitations. First, this study was a retrospective single-center study. The sample size and selection bias were limitations. We divided the patients into four groups according to the ABI and baPWV; however, the sample size of these two groups was very small, potentially biasing some of the results.

**Comment 7:** Fazekas score for each group should be reported in the demographics table.

**Response 7:** Per your suggestion, we have added the Fazekas score in the Tables.

**Comment 8:** In the discussion, the authors mention that baPWV is preferable, but cfPWV has been established as a more robust measure and is the gold standard in the literature. It is also non-invasive. These caveats of baPWV should be acknowledged in the discussion.

**Response 8:** Thank you for the suggestion. We have added the following text:

Lines 280-282

The baPWV is measured between two sites along the arterial system and is preferred as it is easy to perform; however, cfPWV has been established as a more robust measure and is the gold standard.

**Reviewer #2:** The manuscript was substantially revised.

**Comment 1:** “exclud”→”exclude”

In line 72, “…Alzheimer’s pathology. Therefore, we tried to exclud cases with cerebral microbleeds…” should be changed as “…Alzheimer’s pathology. Therefore, we tried to exclude cases with cerebral microbleeds…”?

**Response 1:** We apologize for the typographical error. We have fixed the error.

**Comment 2:** p=0.10
In the methods, the authors described that “a value of p = 0.10 was used to indicate statistical significance.” Is there any literature that could refer to defend the choice of p=0.10?

**Response 2:** For performing the multivariate analyses, we selected the significant factors from the univariate analyses using the cut-off value of p=0.10. In order to select the optimal number of co-variates, we used the cut-off value of p=0.10. There is no fixed rule, but p=0.10 is sometimes used.

Lines 141-143

Univariate analysis was used to investigate the association of MMSE scores with several factors, and a value of p = 0.10 was used to indicate statistical significance for multivariate analysis.
