# Supplementary material for: Association of ankle-brachial index with cognitive decline in patients with lacunar infarction
Source: PLoS One. 2022 Feb 4;17(2):e0263525. doi: 10.1371/journal.pone.0263525 (PMC8815973; doi:10.1371/journal.pone.0263525)
Supplement: Attachment — Submitted filename: response_to_reviewrs_R3_submission.docx [file pone.0263525.s008.docx]

Thank you for reviewing our manuscript. We appreciate your comments and suggestions. We have revised the manuscript in accordance with your comments. Our point-by-point responses to the comments are presented below.

**Response to the reviewers**

**Reviewer #2:** The manuscript was substantially revised, however several questions have been raised.

**Response:** We appreciate your comments and advice. We have responded to each of your comments below and denoted the corresponding revisions in the manuscript in red color.

**Comment 1:** p=0.10
In the methods, the authors described that “Univariate analysis was used to investigate the association of MMSE　scores with several factors, and a value of p = 0.10 was used to indicate statistical significance for multivariate analysis. Subsequently, multivariate analysis was performed with selected factors determined from univariate analysis. " This sentence should be changed something like to "Univariate analysis was used to investigate the association of MMSE scores with several factors. Subsequently, multivariate analysis was performed to estimate and test the independent effects of selected factors on MMSE score. Each of those factors was determined from univariate analysis if the p value was 0.1 or less."

**Response 1:** We appreciate your helpful comment. According to your suggestion, we have revised the manuscript as follows.

Lines 141-144

Univariate analysis was used to investigate the association of MMSE scores with several factors. Subsequently, multivariate analysis was performed to estimate and test the independent effects of selected factors on MMSE score. Each of those factors was determined from univariate analysis if the p value was 0.10 or less.

**Comment 2:** Power analysis
The authors have already declared that "Based on an alpha level of 0.05 and power of 0.80, we estimated that we would require at least 128 participants." However, in the discussion, the sentence "the sample size and selection bias were limitations." was presented. I could not understand this discrepancy.

**Response 2:** We apologize for the confusing expression. We revised as follows.

Line 309

The selection bias was limitation.

**Comment 3:** Bonferroni correction and Tukey’s honestly significant difference (HSD) test
It is hard to understand the sentence "For multiple comparisons, the data were analyzed using one-way analysis of variance (ANOVA), and Bonferroni correction and Tukey’s honestly significant difference (HSD) test were performed." This should be changed to "For multiple comparisons, the data were analyzed using one-way analysis of variance (ANOVA), followed by post-hoc Tukey HSD test, with Bonferroni correction."

**Response 3:** We appreciate your helpful comment. According to your suggestion, we have revised the manuscript as follows.

Lines 146-148

For multiple comparisons, the data were analyzed using one-way analysis of variance (ANOVA), followed by post-hoc Tukey’s honestly significant difference (HSD) test, with Bonferroni correction.
